# ﻿Two new species of *Colletotrichum* (Glomerellaceae, Glomerellales) and a new host record for *Colletotrichum karsti* on *Rosa chinensis* from southwestern China

**DOI:** 10.3897/mycokeys.122.161091

**Published:** 2025-09-18

**Authors:** Chada Norphanphoun, Jia-Ping Wang, Xing-Chang Wang, Herbert Dustin Aumentado, Feng-Quan Liu, Yong Wang

**Affiliations:** 1 Department of Plant Pathology, Agricultural College, Guizhou University, Guiyang, Guizhou 550025, China Guizhou University Guizhou China; 2 Center of Excellence in Fungal Research, Mae Fah Luang University, Chiang Rai 57100, Thailand Mae Fah Luang University Chiang Rai Thailand

**Keywords:** Ascomycota, fungal diversity, fungal morphologically, geographical record, molecular phylogeny, phylogeny, species complex, synonymy, taxonomy

## Abstract

*Colletotrichum*, a genus of ascomycetous fungi in the family Glomerellaceae, includes many important plant pathogens worldwide. In this study, eight fungal strains collected from plant hosts in southwestern China (Guizhou and Yunnan provinces) were identified as four species of *Colletotrichum*. Two novel species, *Colletotrichum actinidicola* and *Colletotrichum poalesicola*, are described based on morphological characteristics and multilocus (ITS, *gapdh*, *chs-1*, *act*, *β-tubulin*, *his3*, and *cal*) phylogenetic analyses. Phylogenetic analyses placed strains of *Colletotrichum fici* in a well-supported clade with *Colletotrichum boninense*, and these taxa are treated as synonyms of *Colletotrichum boninense*. Similarly, *Colletotrichum wuxuhaiense* clustered with *Colletotrichum karsti* and is synonymized with it based on genetic divergence and morphological similarity. Additionally, *Colletotrichum karsti* isolated from *Rosa chinensis* (Rosaceae), is introduced as a new host record. One previously known species, *Colletotrichum boninense*, was also recorded. The integration of molecular and morphological data facilitated accurate species delimitation and improved understanding of *Colletotrichum* diversity in this region. These findings contribute to the growing body of knowledge on the taxonomy, host associations, and distribution of *Colletotrichum* species in Asia.

## ﻿Introduction

*Colletotrichum* is a genus within the phylum Ascomycota, order Glomerellales, and family Glomerellaceae (Hyde et al. 2014). According to Index Fungorum (accessed 14 May 2025), the genus currently includes 1,078 accepted species names, with *C. lineola* designated as the type species. Members of this genus are recognized as important plant pathogens, responsible for anthracnose and *Colletotrichum* blight diseases in a wide range of hosts, such as fruits, vegetables, ornamental plants, and staple crops (Than et al. 2008; Cannon et al. 2012; Huang et al. 2013; Lima et al. 2013; Armand et al. 2023). In addition to their pathogenic roles, some *Colletotrichum* species can exist as endophytes or latent pathogens, residing within host tissues without causing symptoms until the host is stressed or environmental conditions become favorable (Dean et al. 2012; Jayawardena et al. 2016, 2021; Talhinhas and Baroncelli 2021; Hu et al. 2024).

Morphologically, *Colletotrichum* species are characterized by the production of conidia, conidiomata, and setae; however, significant variation exists among species, which contributes to the challenges of accurate identification based on morphological traits (Crouch et al. 2009). The complex pathogenicity, broad host range, and overlapping morphological features of these fungi have historically complicated their classification and disease management (Sutton et al. 1992). However, the advent of molecular tools has significantly improved our understanding of *Colletotrichum* taxonomy and phylogenetic relationships. Molecular approaches, such as multilocus sequence typing (MLST) using markers like the internal transcribed spacer (ITS), actin (*act*), betatubulin (*β-tubulin*), glyceraldehyde-3-phosphate dehydrogenase (*gapdh*), histone H3 (*his3*), chitin synthase 1 (chs-1), and calmodulin (*cal*), have proven essential for accurate species identification and the resolution of cryptic species. Currently, the genus *Colletotrichum* is classified into 15 species complexes, including *C. acutatum*, *C. agaves*, *C. boninense*, *C. caudatum*, *C. dematium*, *C. destructivum*, *C. dracaenophilum*, *C. gigasporum*, *C. gloeosporioides*, *C. graminicola*, *C. magnum*, *C. orbiculare*, *C. orchidearum*, *C. spaethianum*, and *C. truncatum* (Marin-Felix et al. 2017; Damm et al. 2019; Jayawardena et al. 2020; Bhunjun et al. 2021). Among these, the *C. boninense* species complex represents one of the largest complexes within the genus, encompassing more than 40 recognized species (Damm et al. 2012; Lu et al. 2025). This complex has been extensively studied as it includes anthracnose pathogens and endophytes associated with a diverse range of hosts. Accurate identification within this clade relies on multilocus molecular phylogenetic analyses using gene regions such as ITS, *gapdh*, *chs-1*, *actin*, *β-tubulin*, *his3*, and *cal* (Damm et al. 2012; Jayawardena et al. 2020; Bhunjun et al. 2021). In this study, eight *Colletotrichum* strains collected from southeastern China were examined. The primary aim was to identify these isolates based on a combination of morphological characteristics and multilocus phylogenetic data, to confirm their taxonomic placement and novel ecological associations within these diverse ecosystems.

## ﻿Materials and methods

### ﻿Sampling and examination of specimens

Samples were collected from 2023 to 2024, including symptomatic leaves of *Actinidia chinensis*, *Poaceae* sp., and *Rosa chinensis* from Guizhou Province, and symptomatic fruit of *Juglans regia* L from Yunnan Province, China. Fresh specimens were collected, tagged (Rathnayaka et al. 2024), and stored in paper bags; they were then examined and described in the laboratory. Macro-morphological characteristics were examined using a SMZ-T4 stereoscopic microscope (Chongqing, China). Micro-morphological characteristics (conidiomata, conidiophores, conidiogenous cells, and conidia) were studied using a ZEISS Axioscope 5 microscope (Jena, Germany) and photographed by using an AxioCam 208 color camera (Carl Zeiss Microscopy GmbH, Jena, Germany), while the size measurements (conidiophores, conidiogenous cells, and conidia) were taken with the assistance of ZEN 3.0 (Blue Edition) software (Jena, Germany). Photoplates were made using Adobe Photoshop 2025 version 26.5 (Adobe Systems, CA, USA).

The cultures were acquired by the tissue isolation technique as described in the study of Norphanphoun et al. (2018). Single hyphal tips were transferred onto 2% potato agar (PA) plates at room temperature (25 °C ± 2 °C) for one week, with a 12-hour dark and 12-hour light cycle. The cultural features were observed and documented at 5, 7, and 14 days. The morphological characteristics of the culture were analyzed during the entire cultivation duration. Pure cultures were cultivated on Potato Dextrose Agar (PDA) for further experiments. Dried cultures were prepared as described by Sinclair and Dhingra (1995) and deposited at the Herbarium of the Department of Plant Pathology, Agricultural College, Guizhou University (HGUP). Living cultures have been deposited in the culture collection at the Plant Pathology Department of the College of Agriculture, Guizhou University, China (GUCC). The enumeration for the new taxon was conducted in the MycoBank online database (https://www.mycobank.org; Robert et al. 2013).

### ﻿DNA extraction, amplification via PCR, and sequencing

Genomic DNA was extracted from fresh fungal mycelia grown on PDA at room temperature (25 °C ± 2) for two weeks using the Biospin Fungal Genomic DNA Extraction Kit (BioFlux) following the manufacturer’s protocols. Polymerase chain reactions (PCR) were carried out using the following primer pairs: ITS5/ITS4 to amplify the internal transcribed spacer region (ITS), ACT512F/ACT738R for actin (*act*), GDF/GDR for partial glyceraldehyde-3-phosphate dehydrogenase region (*gapdh*), T1/ Btub4Rd or Bt2a/Bt2b for beta-tubulin (*β-tubulin*), CHS-79F/ CHS-354R for chitin synthase (*chs-1*), H3F/H3R for imidazoleglycerol-phosphate dehydratase (*his3*), CL1C/CL2C for calmodulin (*cal*) (Kim et al. 2021; Li et al. 2021; Schena et al. 2014; Weir et al. 2012).

The amplification reactions were carried out using the following protocol: a 20μl reaction volume containing 1 μl of DNA template, 1 μl (20 μM stock concentration) of each forward and reverse primer, 10 μl of 2Mix (Vazyme Biotech Co., Ltd.), and 7 μl of double-distilled water (ddH_2_O). The PCR thermal cycling program for each locus is described in Table 1. The purification and sequencing of PCR products using the amplification primers specified above were conducted at Sangon Biotech (Shanghai, China) Co., Ltd. for Sanger sequencing. After sequencing, the sequence data were uploaded to GenBank, and the relevant information is listed in Table 2.

**Table 1.** Polymerase chain reactions (PCR) thermal cycling programs for each locus.

Gene	Primers	PCR thermal cycle protocols*
ITS	ITS1/ITS4	ID 95 °C for 5 min, 35 cycles of D at 95 °C for 30 s, A at 52 °C for 30 s, E at 72 °C for 1 min, FE at 72 °C for 10 min
*actin*	ACT512F/ACT738R	ID 95 °C for 5 min, 35 cycles of D at 95 °C for 30 s, A at 60.3 °C for 30 s, E at 72 °C for 1 min, FE at 72 °C for 10 min
*gapdh*	GDF/GDR	ID 95 °C for 5 min, 35 cycles of D at 95 °C for 30 s, A at 61.9 °C for 30 s, E at 72 °C for 1 min, FE at 72 °C for 10 min
*β-tubulin*	T1/T2	ID 95 °C for 5 min, 35 cycles of D at 95 °C for 30 s, A at 55.7 °C for 30 s, E at 72 °C for 1 min, FE at 72 °C for 10 min
	2a/2b	ID 95 °C for 5 min, 35 cycles of D at 95 °C for 30 s, A at 61.3 °C for 30 s, E at 72 °C for 1 min, FE at 72 °C for 10 min
*chs-1*	CHS-79F/ CHS-354R	ID 95 °C for 5 min, 35 cycles of D at 95 °C for 30 s, A at 59.4 °C for 30 s, E at 72 °C for 1 min, FE at 72 °C for 10 min
*his3*	H3F/H3R	ID 95 °C for 5 min, 35 cycles of D at 95 °C for 30 s, A at 62.1 °C for 30 s, E at 72 °C for 1 min, FE at 72 °C for 10 min
*cal*	CL1C/CL2C	ID 95 °C for 5 min, 35 cycles of D at 95 °C for 30 s, A at 54.6 °C for 30 s, E at 72 °C for 1 min, FE at 72 °C for 10 min

*ID: initial denaturation; D = denaturation; A = annealing; E = elongation; FE = final extension.

**Table 2.** GenBank accession numbers of the sequences used in phylogenetic analyses.

Species	Strain no.	Host	Location	Gene Bank accession number							References
				ITS	*gapdh*	*chs-1*	*act*	*β-tubulin*	*his*	*cal*	
***C. actinidicola* sp. nov.**	**GUCC 25-0036 ^T^**	***Actinidia chinensis***	**China**	**PV771185**	**PV775956**	**PV775948**	**PV775940**	**PV775980**	**PV775964**	–	**In this study**
***C. actinidicola* sp. nov.**	**GUCC 25-0058**	***Actinidia chinensis***	**China**	**PV771186**	**PV775957**	**PV775949**	**PV775941**	**PV775981**	**PV775965**	–	**In this study**
*C. annellatum*	CBS 129826 ^T^	*Hevea brasiliensis*, leaf	Colombia	JQ005222	JQ005309	JQ005396	JQ005570	JQ005656	JQ005483	JQ005743	Damm et al. (2012)
*C. araujiae*	BBB:Ah35-04 ^T^	*Araujia hortorum*, leaf, stem and fruit	Argentina	OP035058	OP067659	–	–	OP067660	–	–	Tan et al. (2022)
*C. beeveri*	CBS 128527 ^T^	*Brachyglottis repanda*	New Zealand	JQ005171	JQ005258	JQ005345	JQ005519	JQ005605	JQ005432	JQ005692	Damm et al. (2012)
***C. boninense***	**GUCC 25-0020**	***Juglans regia***	**China**	**PV771187**	**PV775958**	**PV775950**	**PV775942**	**PV775982**	**PV775966**	–	**In this study**
***C. boninense***	**GUCC 25-0059**	***Juglans regia***	**China**	**PV771188**	**PV775959**	**PV775951**	**PV775943**	**PV775983**	**PV775967**	–	**In this study**
*C. boninense*	CBS 123755 ^T^	Crinum asiaticum var. sinicum	Japan	JQ005153	JQ005240	JQ005327	JQ005501	JQ005588	JQ005414	JQ005674	Damm et al. (2012)
*C. boninense*	CBS 123756	Crinum asiaticum var. sinicum	Japan	JQ005154	JQ005241	JQ005328	JQ005502	JQ005589	JQ005415	JQ005675	Damm et al. (2012)
*C. boninense*	CGMCC 3.15168	*Bletilla ochracea*, leaf	China	KC244165	KC843491	–	KC843554	KC244158	–	–	Tao et al. (2013)
*C. boninense*	MFLUCC 14-0124	*Dendrobium* sp., stem	Thailand	MG792809	MK165700	–	–	MH351286	–	–	Ma et al. (2018)
*C. boninense*	MFLUCC 14-0086	*Dendrobium* sp., leaf	Thailand	MG792816	MH673668	–	MH376390	MH351281	–	–	Ma et al. (2018)
*C. boninense*	CBS 128547, ICMP 10338	*Camellia* sp.	New Zealand	JQ005159	JQ005246	JQ005333	JQ005507	JQ005593	JQ005420	JQ005680	Damm et al. (2012)
*C. boninense*	MAFF 306162, ICMP 18596	Crinum asiaticum var. sinicum, leaf	Japan	JQ005155	JQ005242	JQ005329	JQ005503	–	JQ005416	JQ005676	Damm et al. (2012)
*C. boninense*	CBS 112115, STE-U 2966	*Leucospermum* sp.	Australia	JQ005160	JQ005247	JQ005334	JQ005508	JQ005594	JQ005421	JQ005681	Damm et al. (2012)
*C. boninense*	CBS 129831, STE-U 2965	*Leucospermum* sp.	Australia	JQ005161	JQ005248	JQ005335	JQ005509	JQ005595	JQ005422	JQ005682	Damm et al. (2012)
*C. boninense*	CBS 128549, ICMP 15444	*Solanum betaceum*, flowers	New Zealand	JQ005156	JQ005243	JQ005330	JQ005504	JQ005590	JQ005417	JQ005677	Damm et al. (2012)
*C. boninense*	CBS 128506, ICMP 12950	*Solanum lycopersicum*, fruit rot	New Zealand	JQ005157	JQ005244	JQ005331	JQ005505	JQ005591	JQ005418	JQ005678	Damm et al. (2012)
*C. boninense*	CBS 128546, ICMP 18595	*Tecomanthe speciosa*	New Zealand	JQ005158	JQ005245	JQ005332	JQ005506	JQ005592	JQ005419	JQ005679	Damm et al. (2012)
*C. boninense* (=*C. fici*)	MFLU 18-2615 ^T^	*Ficus ampelas*, leaf	China	MW114364	–	MW177698	MW151582	–	–	–	Tennakoon et al. (2021)
*C. boninense* (=*C. fici*)	NCYUCC 19-0336	*Ficus ampelas*, leaf	China	MW114365	–	MW177699	MW151583	–	–	–	Tennakoon et al. (2021)
*C. boninense* (=*C. fici*)	NCYUCC 19-0337	*Ficus ampelas*, leaf	China	MW114366	–	MW177700	MW151584	–	–	–	Tennakoon et al. (2021)
*C. brasiliense*	CBS 128501 ^T^	*Passiflora edulis*, fruit anthracnose	Brazil	JQ005235	JQ005322	JQ005409	JQ005583	JQ005669	JQ005496	JQ005756	Damm et al. (2012)
*C. brassicicola*	CBS 101059 ^T^	Brassica oleracea var. gemmifera, leaf spot	New Zealand	JQ005172	JQ005259	JQ005346	JQ005520	JQ005606	JQ005433	JQ005693	Damm et al. (2012)
*C. bromeliacearum*	LC0951 ^T^	* Bromeliaceae *	China	MZ595832	MZ664077	MZ799267	MZ664130	MZ673956	MZ673843	–	Liu et al. (2022)
*C. camelliae*-*japonicae*	CGMCC 3.18118 ^T^	*Camellia japonica*	Japan	KX853165	KX893584	–	KX893576	KX893580	MZ673859	–	Hou et al. (2016)
*C. capsicicola*	PC159	*Capsicum annuum*, leaf	Brazil	OR505853	OR599648	OR599656	OR599652	–	–	–	Oliveira et al. (2024)
*C. capsicicola*	URM 8822	*Capsicum annuum*, leaf	Brazil	OR505852	OR599647	OR599655	OR599651	–	–	–	Oliveira et al. (2024)
*C. catinaense*	CBS 142417 ^T^	*Citrus reticulata*, leaf	Italy	KY856400	KY856224	KY856136	KY855971	KY856482	KY856307	KY856053	Guarnaccia et al. (2017)
*C. celtidis*	MFLUCC 20-0177 ^T^	Celtis formosana, leaf	China	MW114362	–	MW177696	MW151580	MW148274	–	–	Tennakoon et al. (2021)
*C. celtidis*	NCYUCC 19-0335	Celtis formosana, leaf	China	MW114363	–	MW177697	MW151581	MW148275	–	–	Tennakoon et al. (2021)
*C. chamaedoreae*	LC13868 ^T^	*Chamaedorea erumpens*, leaf	China	MZ595890	MZ664084	MZ799274	MZ664188	MZ674008	MZ673910	–	Liu et al. (2022)
*C. chongqingense*	CS0612 ^T^	Tea	China	MG602060	MG602022	MT976117	MT976107	MG602044	–	–	Wan et al. (2021)
*C. citricola*	CBS 134228 ^T^	*Citrus unshiu*	China	KC293576	KC293736	KY856140	KC293616	KC293656	KY856311	KC293696	Hou et al. (2016)
*C. cliviigenum*	CBS 146825	*Clivia* sp., leaf	South Africa	MZ064415	MZ078178	MZ078161	MZ078143	MZ078260	MZ078180	–	Crous et al. (2021)
*C. colombiense*	CBS 129818 ^T^	*Passiflora edulis*, leaf	Colombia	JQ005174	JQ005261	JQ005348	JQ005522	JQ005608	JQ005435	JQ005695	Damm et al. (2012)
*C. condaoense*	CBS 134299 ^T^	*Ipomoea pes*-*caprae*, leaf	Vietnam	MH229914	MH229920	MH229926	–	MH229923	MH229927	–	Crous et al. (2018)
*C. constrictum*	CBS 128504 ^T^	*Citrus limon*, fruit rot	New Zealand	JQ005238	JQ005325	JQ005412	JQ005586	JQ005672	KY856313	JQ005759	Damm et al. (2012)
*C. cymbidiicola*	IMI 347923 ^T^	*Cymbidium* sp., leaf lesion	Australia	JQ005166	JQ005253	JQ005340	JQ005514	JQ005600	JQ005427	JQ005687	Damm et al. (2012)
*C. dacrycarpi*	CBS 130241 ^T^	*Dacrycarpus dacrydioides*, leaf endophyte	New Zealand	JQ005236	JQ005323	JQ005410	JQ005584	JQ005670	JQ005497	JQ005757	Damm et al. (2012)
*C. diversum*	LC11292 ^T^	*Philodendron selloum*	China	MZ595844	MZ664081	MZ799272	MZ664142	MZ673965	MZ673864	–	Liu et al. (2022)
*C. doitungense*	MFLUCC 14-0128 ^T^	*Dendrobium* sp., leaf	Thailand	MF448524	MH049480	–	MH376385	MH351277	–	–	Ma et al. (2018)
*C. feijoicola*	CBS 144633 ^T^	*Acca sellowiana*, leaf	Portugal	MK876413	MK876475	–	MK876466	MK876507	–	–	Crous et al. (2019)
*C. hippeastri*	CBS 125376 T ^T^	*Hippeastrum vittatum*, leaf	China	JQ005231	JQ005318	JQ005405	JQ005579	JQ005665	JQ005492	JQ005752	Damm et al. (2012)
***C. karsti***	**GUCC 25-0037**	***Rosa chinensis***	**China**	**PV771183**	**PV775954**	**PV775946**	**PV775938**	**PV775978**	**PV775962**	–	**In this study**
***C. karsti***	**GUCC 25-0060**	***Rosa chinensis***	**China**	**PV771184**	**PV775955**	**PV775947**	**PV775939**	**PV775979**	**PV775963**	–	**In this study**
*C. karsti*	CORCG6 ^T^	*Vanda* sp., leaf	China	HM585409	HM585391	HM582023	HM581995	HM585428	–	HM582013	Yang et al. (2011)
*C. karsti*	*CORCKl*	*Calanthe argenteo*-*striata*	China	HM585406	HM585387	HM582019	HM581991	HM585424	–	HM582010	Yang et al. (2011)
*C. karsti*	*CORCK3*	*Eria coronaria*	China	HM585407	HM585388	HM582020	HM581992	HM585427	–	HM582011	Yang et al. (2011)
*C. karsti*	*CORCS4*	*Pleione bulbocodioides*	China	HM585405	HM585390	HM582022	HM581994	HM585426	–	HM582012	Yang et al. (2011)
*C. karsti*	*CORCX7*	*Arundina graminifolia*	China	HM585408	HM585389	HM582021	HM581993	HM585425	–	HM582009	Yang et al. (2011)
*C. karsti*	CBS 110779	*Eucalyptus grandis*	South Africa	JQ005198	JQ005285	JQ005372	JQ005546	JQ005632	JQ005459	JQ005719	Damm et al. (2012)
*C. karsti*	CBS 127597	*Diospyros australis*	Australia	JQ005204	JQ005291	JQ005378	JQ005552	JQ005638	JQ005465	JQ005725	Damm et al. (2012)
*C. karsti* (=*C. wuxuhaiense*)	YMF 1.04951 ^T^	*Potamogeton crispus*, root	China	OL842173	OL981268	OL981294	OL981242	OL981228	–	–	Zheng et al. (2022)
*C. karsti* (=*C. wuxuhaiense*)	F34	*Potamogeton pectinatus*, root	China	OL842175	OL981270	OL981296	OL981244	OL981230	–	–	Zheng et al. (2022)
*C. laurosilvaticum*	RGM 3406 ^T^	*Laurelia sempervirens*, leaf	Chile	OR644582	OR644989	OR645042	OR645095	OR645147	OR659720	–	Zapata et al. (2024)
*C. limonicola*	CBS 142410 ^T^	*Citrus limon*, leaf	Malta	KY856472	KY856296	KY856213	KY856045	KY856554	KY856388	KY856125	Guarnaccia et al. (2017)
*C. novae*-*zelandiae*	CBS 128505 ^T^	*Capsicum annuum*	New Zealand	JQ005228	JQ005315	JQ005402	JQ005576	JQ005662	JQ005489	JQ005749	Hou et al. (2016)
*C. oncidii*	CBS 129828 ^T^	*Oncidium* sp., leaf	Germany	JQ005169	JQ005256	JQ005343	JQ005517	JQ005603	JQ005430	JQ005690	Damm et al. (2012)
*C. palki*	RGM 3055 ^T^	*Cestrum parqui*, leaf	Chile	OR644584	OR644991	OR645044	OR645097	OR645149	OR659722	–	Zapata et al. (2024)
*C. parsonsiae*	CBS 128525 ^T^	*Parsonsia capsularis*, leaf endophyte	New Zealand	JQ005233	JQ005320	JQ005407	JQ005581	JQ005667	JQ005494	JQ005754	Damm et al. (2012)
*C. pernambucoense*	PC86	*Capsicum annuum*, leaf	Brazil	OR505855	OR599650	OR599658	OR599654	–	–	–	Oliveira et al. (2024)
*C. pernambucoense*	URM 8821	*Capsicum annuum*, leaf	Brazil	OR505854	OR599649	OR599657	OR599653	–	–	–	Oliveira et al. (2024)
*C. petchii*	CBS 378.94 ^T^	*Dracaena marginata*, spotted leaves	Italy	JQ005223	JQ005310	JQ005397	JQ005571	JQ005657	JQ005484	JQ005744	Damm et al. (2012)
***C. poalesicola* sp. nov.**	**GUCC 25-0040 ^T^**	** Poales **	**China**	**PV771181**	**PV775952**	**PV775944**	**PV775936**	**PV775976**	**PV775960**	–	**In this study**
***C. poalesicola* sp. nov.**	**GUCC 25-0057**	** Poales **	**China**	**PV771182**	**PV775953**	**PV775945**	**PV775937**	**PV775977**	**PV775961**	–	**In this study**
*C. philodendricola*	CGMCC 3.19290 ^T^	*Philodendron tatei* cv. Congo, leaf	China	MH105257	MH105261	MH105265	MH105273	MH105277	MH105269	MH105281	Xue et al. (2020)
*C. philodendricola*	LZJZ4	*Philodendron tatei* cv. Congo, leaf	China	MH105260	MH105264	MH105268	MH105276	MH105280	MH105272	MH105284	Xue et al. (2020)
*C. phyllanthi*	CBS 175.67 ^T^	*Phyllanthus acidus*, anthracnose	India	JQ005221	JQ005308	JQ005395	JQ005569	JQ005655	JQ005482	JQ005742	Damm et al. (2012)
*C. pseudoboninense*	CGMCC 3.19755 ^T^	*Philodendron tatei* cv. Congo, leaf	China	MK796540	MK796573	–	MK796547	MK796554	MK796580	–	Xue et al. (2020)
*C. spicati*	YMF 1.04942 ^T^	*Myriophyllum spicatum*, stem	China	OL842171	OL981266	OL981292	OL981240	OL981226	–	–	Zheng et al. (2022)
*C. spicati*	F6	*Potamogeton wrightii*, leaf	China	OL842172	OL981267	OL981293	OL981241	OL981227	–	–	Zheng et al. (2022)
*C. torulosum*	CBS 128544 T	*Solanum melongena*	New Zealand	JQ005164	JQ005251	JQ005338	JQ005512	JQ005598	JQ005425	JQ005685	Damm et al. (2012)
*C. watphraense*	MFLUCC 14-0123 ^T^	*Dendrobium* sp., stem	Thailand	MF448523	MH049479	–	MH376384	MH351276	–	–	Ma et al. (2018)
*C. agaves* (outgroup)	LC0947	*Agave* sp.	Thailand	MZ595831	MZ664053	MZ799266	MZ664129	MZ673955	MZ673842	–	Liu et al. (2022)
*C. euphorbiae* (outgroup)	CBS 134725 ^T^	*Euphorbia* sp., leaf	South Africa	KF777146	KF777131	KF777128	KF777125	KF777247	KF777134	–	Crous et al. (2013)

Ex-type/ex-epitype/ex-neotype/ex-lectotype strains are marked with ^T^; CBS CBS-KNAW Fungal Biodiversity Centre, Utrecht, The Netherlands; CGMCC China General Microbiological Culture Collection Center; GUCC the Plant Pathology Department of the College of Agriculture, Guizhou University, China; ICMP International Collection of Microorganisms from Plants; IMI International Mycological Institute; LC the LC Culture Collection (a personal culture collection of Lei Cai, housed in the Institute of Microbiology, Chinese Academy of Sciences); MAFF the Genetic Resources Center, National Agriculture and Food Research Organization, Tsukuba, Ibaraki, Japan; MFLU Mae Fah Luang University Herbarium Collection, Chiang Rai, Thailand; MFLUCC Mae Fah Luang University Culture Collection, Chiang Rai, Thailand; NTUCC the Department of Plant Pathology and Microbiology, National Taiwan University Culture Collection; RGM the Chilean Collection of Microbial Genetic Resources; STE-U Culture Collection of the Department of Plant Pathology, University of Stellenbosch, South Africa; URM the University Recife Mycology Culture Collection of the Universidade Federal de Pernambuco, Brazil; YMF the Herbarium of the Laboratory for Conservation and Utilization of Bio-Resources, Yunnan University, Kunming, Yunnan, China. The strains in this study are in bold. Missing data is indicated by ‘–’.

### ﻿Phylogenetic analysis

The raw readings were processed and organized into contigs using Geneious Prime 2025.0.3 Java Version 11.0.24+8 (64-bit) software (http://www.geneious.com). The newly generated sequences were utilized as queries to conduct a BLASTn search against the non-redundant (nr) database in GenBank. The retrieval of similar sequences was conducted, followed by the construction of numerous alignments. The GenBank taxonomy browser was utilized to verify all sequences classified as *Colletotrichum* in the database. BioEdit version 7.2.5 (Hall 1999) was used to assign open reading frames of the protein-coding sequences of *gapdh*, *chs-1*, *actin*, *β-tubulin*, *his3*, and *cal* according to reference sequences in the GenBank database. The combined sequence data from all loci (ITS, *gapdh*, *chs-1*, *act*, *β-tubulin*, *his*, *cal*) were used to perform maximum likelihood (ML), maximum parsimony (MP), and Bayesian inference (BI) analysis.

The dataset for each gene region was independently aligned using the ‘auto’ strategy (based on data size) in MAFFT (Katoh et al. 2019) and trimmed with the ‘gappyout’ method (based on gaps’ distribution) in TrimAl (Capella-Gutiérrez et al. 2009). BioEdit v. 7.0.9.0 (Hall 1999) was utilized for manual editing, where needed. The best-fit nucleotide substitution models for each dataset were selected based on the Bayesian information criterion (BIC) with rate heterogeneity, as determined by ModelFinder (Kalyaanamoorthy et al. 2017). Afterwards, all datasets were concatenated with partition information for the subsequent phylogenetic analyses.

Maximum likelihood (ML), maximum parsimony (MP), and Bayesian posterior probability analysis (PP) were performed using the CIPRES Science Gateway (https://www.phylo.org/portal2) (Miller et al. 2010). The maximum parsimony phylogenetic tree was performed using PAUP XSEDE (Swofford 2002). The maximum likelihood tree was constructed using RAxMLHPC2 on XSEDE with 1,000 bootstrapping replicates. The ML analyses utilized the GTR + GAMMA model. The Bayesian posterior probability (PP) analysis employed a Markov Chain Monte Carlo (MCMC) algorithm with MrBayes on XSEDE, involving four MCMC chains that ran for 1,000,000 generations and sampled at intervals of 100 generations. The first 25% of constructed trees were eliminated as burn-in, and the remaining trees were used to compute posterior probabilities (Ronquist et al. 2012). The resultant phylograms were visualised with FigTree v. 1.4.4 (Rambaut 2018) and formatted using Adobe Illustrator CC 22.0.0 (Adobe Systems, USA).

The Genealogical Concordance Phylogenetic Species Recognition (GCPSR) model, with a pairwise homoplasy index (PHI) test, was used to analyze the newly generated taxon and its most phylogenetically close neighbors (Quaedvlieg et al. 2014). The PHI test was performed in SplitsTree v. 4.14.6 (Huson 1998; Huson and Bryant 2006) with a concatenated dataset to determine the recombination level among phylogenetically closely related species. A pairwise homoplasy index below a 0.05 threshold (Φw < 0.05) indicated the presence of significant recombination in the dataset. The relationship between closely related species was visualized by constructing a split gap.

## ﻿Results

**Figure 1.**
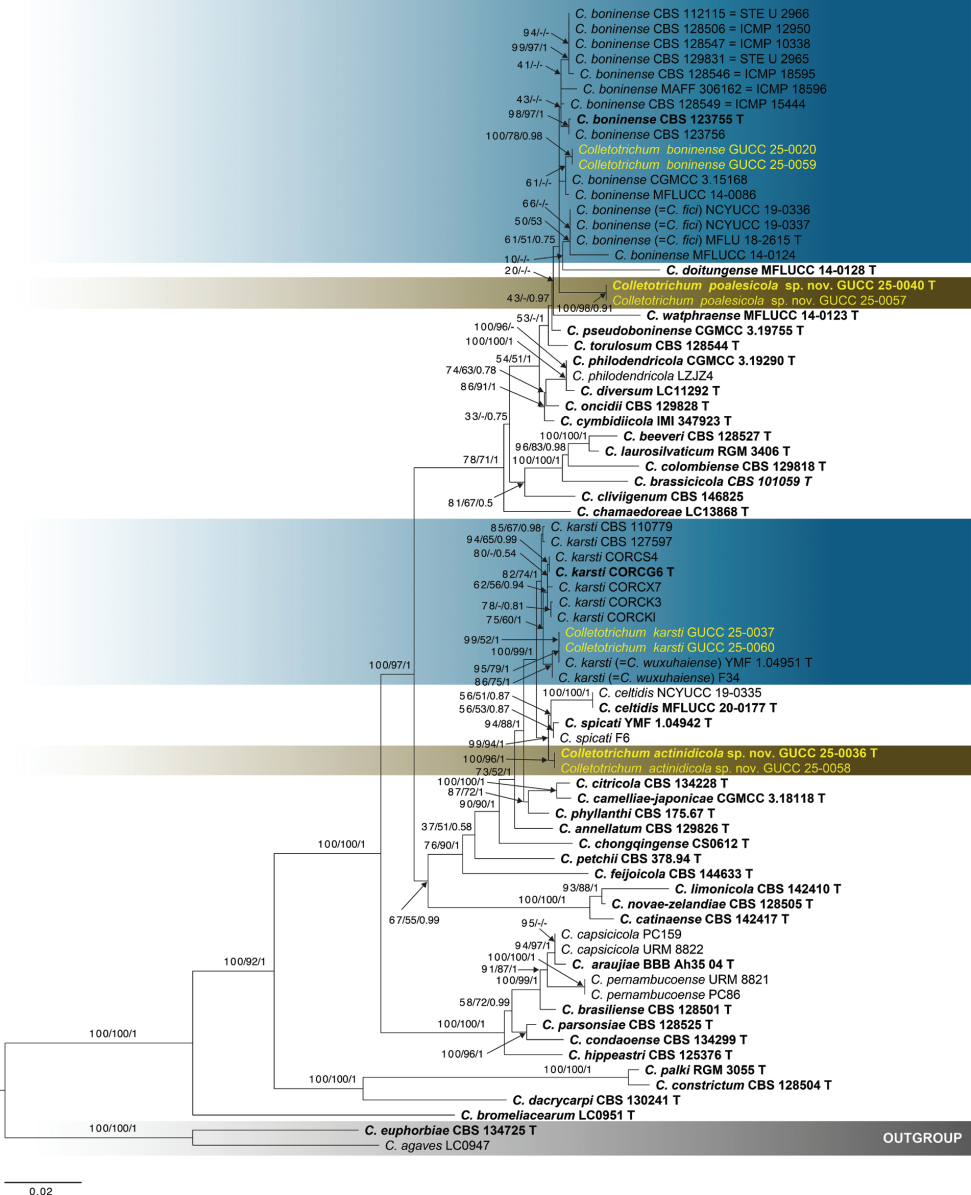
Phylogenetic tree constructed using a maximum likelihood (ML) analysis based on a combined ITS, *gapdh*, *chs*-*1*, *actin*, *β-tubulin*, *his3*, and *cal* sequences, representing *Colletotrichum boninense* species complex. The tree topology of the ML analysis was identical to the Maximum Parsimony (MP) and Bayesian posterior probability (PP) analyses. The final RAxML tree with a likelihood value of –14964.299156 is presented here. The evolutionary model GTR+GAMMA was applied to all the genes. The analysis included seventy (76) taxa with a total of 3,112 characters, with 1,194 distinct alignment patterns, and 29.37% were gaps and undetermined characters. Bootstrap support values for BSML and BSMP, and Bayesian Posterior Probabilities (PPBI) are indicated at the nodes as BSML/BSMP/PPBI. The tree is rooted with *C. agaves* (LC0947) and *C. euphorbiae* (CBS 134725). Type strains are denoted in bold and T, sequences generated in this study are in yellow. Bar = 0.02 represents the estimated number of nucleotide substitutions per site per branch.

To evaluate tree topology and clade support, single-locus phylogenetic analyses were initially conducted prior to constructing the multilocus combined dataset. This study introduces two novel *Colletotrichum* species and reports a new host record for a known species, with one previously identified species being reported. Phylogenetic relationships were inferred using a combined dataset of seven genetic loci (ITS, *gapdh*, *chs-1*, *act*, *β-tubulin*, *his3*, and *cal*) comprising 68 strains within the *Colletotrichum boninense* species complex. *Colletotrichum agaves* (LC0947) and *C. euphorbiae* (CBS 134725) were designated as outgroup taxa. The concatenated alignment consisted of 2,670 characters, including gaps, partitioned as follows: ITS (1–577), *gapdh* (578–881), *chs-1* (882–1172), *act* (1173–1451), *β-tubulin* (1452–1978), *his3* (1979–2374), and *cal* (2375–3112). Phylogenetic trees were reconstructed using Maximum Likelihood (ML), Maximum Parsimony (MP), and Bayesian Inference (BI) methods. All three approaches yielded congruent topologies, with no significant conflict among the trees. The best-scoring ML tree (Fig. 1) had a final likelihood score of –14964.299156. In this phylogeny, all newly collected strains were clustered within the *C. boninense* species complex. This species complex was separated from the outgroup with strong statistical support (BSML = 100%, BSMP = 100%, PPBI = 1.0), confirming its monophyly and the correct placement of the new strains within this clade. Two newly described taxa, *C. actinidicola* (GUCC 25-0036, GUCC 25-0058) and *C. poalesicola* (GUCC 25-0040, GUCC 25-0057), formed distinct, well-supported lineages. *Colletotrichum actinidicola* was resolved as a sister lineage to *C. spicati* and *C. celtidis*, with robust support values (BSML = 99%, BSMP = 94%, PPBI = 1.0). *Colletotrichum poalesicola* also formed a distinct clade, although it received relatively lower bootstrap support in (BSML = 61%, BSMP = 51%, PPBI = 0.75). Two isolates obtained from *Rosa chinensis* in Yunnan Province (GUCC 25-0060 and GUCC 25-0037) clustered with *C. karsti*, supported by BSML = 82%, BSMP = 74%, and PPBI = 1.0, representing a new host association for this species. Additionally, two isolates (GUCC 25-0020 and GUCC 25-0059) were identified as a distinct strain of *C. boninense*, based on their placement within the complex. The tree topology derived from the multilocus dataset was consistent with those obtained from individual gene trees, reaffirming the stability of phylogenetic placements across analytical methods.

**Figure 2.**
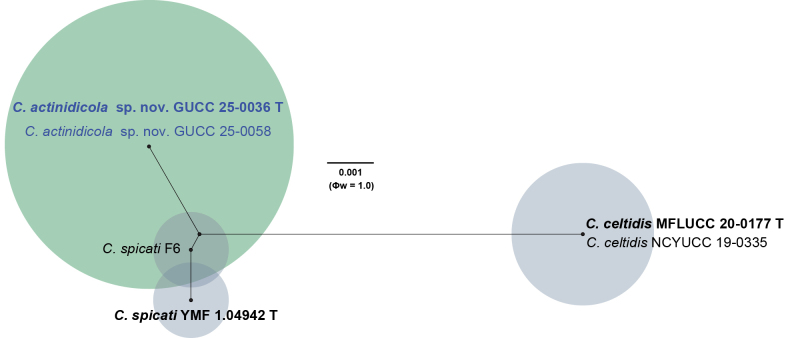
Split graph displaying the results of the pairwise homoplasy index (PHI) test of *Colletotrichum actinidicola* sp. nov and closely related taxa using Log-det transformation and splits decomposition options. Φw ≥ 0.05 from the PHI test signifies no substantial recombination in the dataset. The type species are indicated in bold, and the newly identified taxon and isolates are displayed in blue.

To further validate the taxonomic independence of the novel taxa, the Genealogical Concordance Phylogenetic Species Recognition (GCPSR) criterion was applied. The Pairwise Homoplasy Index (PHI) test was used to detect recombination events among closely related taxa. A Φw value below 0.05 indicates significant recombination, whereas values above this threshold support genetic separation. For both *C. actinidicola* and *C. poalesicola*, the PHI test returned non-significant values (Φw = 1.0), indicating no detectable recombination with closely related species, including *C. spicati*, *C. celtidis*, *C. fici*, and *C. boninense* (Figs 2, 4). These results support the recognition of *C. actinidicola* and *C. poalesicola* as phylogenetically and evolutionarily distinct species.

### ﻿Taxonomy

#### ﻿ Colletotrichum actinidicola

Taxon classificationFungiGlomerellaceae

Norph. & J.P. Wang sp. nov.

3D00F42A-42BD-5B6A-9C18-D73068582882

MycoBank No: 903941

Figs 2, 3

##### Etymology.

The epithet refers to “dweller on *Actinidia*,” utilizing the Latin suffix -cola, meaning “inhabitant” or “dweller.”

##### Type.

China • Guizhou Province, Tongren, Symptomatic leaves of *Actinidia chinensis* (Actinidiaceae), 2024.03.02, coll. Wang Jiaping, TRM2-1/GZ24/GZ24-2 (dried culture, HGUP 25-0043, holotype), ex-type living culture GUCC 25-0036.

##### Description.

Isolated from leaf spot of *Actinidia chinensis* L. ***Sexual morph***: undetermined. ***Asexual morph*: *Conidiomata*** pycnidial, globose, dark brown, superficial on PDA, releasing conidia in a yellow mass, slimy, globose. ***Conidiophores*** produce conidiogenous cells either directly from hyphae or from a cushion of spherical hyaline cells, septate, branched. ***Conidiogenous cells*** hyaline, cylindrical to clavate, straight to flask-shaped, (13.5–)14–40(–41) × (3–)3.3–5(–5.2) μm (mean ± SD = 22 ± 0.7 × 4 ± 0.4 μm). ***Setae*** not observed. ***Conidia*** (10–)14–16(–18) × (5–)5.5–6(–7.2) μm (mean ± SD = 16 ± 1 × 6 ± 1 μm), n = 35, L/W ratio = 2.1, hyaline, aseptate, smooth-walled, ellipsoidal to cylindrical, one end rounded and one end acute or both ends rounded, guttulate, granular. ***Appressoria*** single in short chains, pale brown, thick-walled, entire edge, rarely lobate, smooth-walled.

##### Culture characteristics.

**Figure 3.**
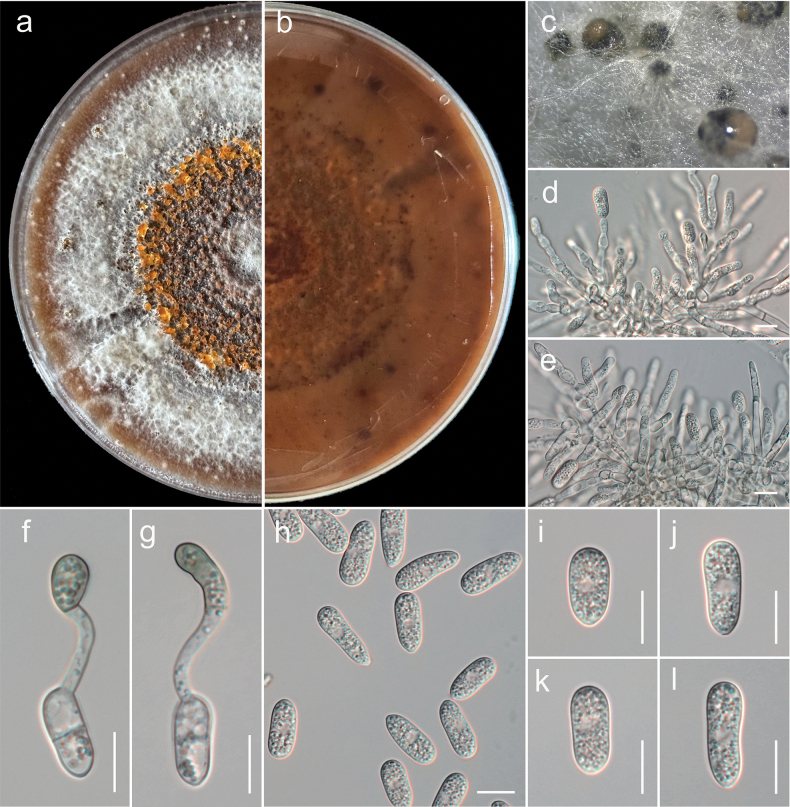
*Colletotrichum actinidicola* (GUCC 25-0020, ex-type). a, b. Culture on PDA (a-above, b-reverse); c. Conidiomata on PDA; d, e. Conidiophore, conidiogenous cells giving rise to conidia; f, g. Appressorium; h–l. Conidia. Scale bars: 10 µm (d–l).

Colonies on MEA reach 7–9 cm in diameter after 7 days at room temperature (±25 °C), exposed to 12 hours of light and 12 hours of darkness. The colonies are rhizoid to filamentous, dense, with a flat or raised surface and a filiform margin. After 14 days, the colony appears white from above, producing grouped pycnidia with an orange ring conidial mass in the center and a white to yellow reverse (Fig. 3a, b).

##### Material examined.

China • Guizhou Province, Tongren, Symptomatic leaves of *Actinidia chinensis* (Actinidiaceae), 22024.03.02, coll. Wang Jiaping, TRM2-1/GZ24/GZ24-2 (dried culture, HGUP 25-0043, holotype), ex-type living culture GUCC 25-0036, GUCC 25-0058.

##### Notes.

Based on a multi-locus phylogenetic analysis, *Colletotrichum actinidicola* is placed within the *C. boninense* species complex, forming a well-supported and distinct lineage closely related to *C. celtidis* and *C. spicati* (Fig. 1). This placement is strongly supported by high bootstrap and posterior probability values (99% BSML, 94% BSMP, and 1.00 PPBI). Morphologically, *C. actinidicola* exhibits key diagnostic features typical of the *C. boninense* complex, including hyaline, smooth-walled conidia with a prominent basal scar and conidiogenous cells exhibiting distinct periclinal thickening (Damm et al. 2012). Despite these similarities, *C. actinidicola* can be distinguished from its closest relatives by conidial morphology and genetic divergence. It produces hyaline, ellipsoidal to cylindrical conidia, measuring (10–)14–16(–18) × (5–)5.5–6(–7.2) µm, whereas *C. celtidis* produces pale brown, straight to slightly curved, broadly ellipsoidal to ovoid conidia with both ends acute, measuring 13–17 × 5–6.5 μm (Tennakoon et al. 2021). In comparison, *C. spicati* possesses wider conidia, measuring 10.9–15.7 × 5.0–8.2 µm (Zheng et al. 2022). Furthermore, pairwise comparisons of nucleotide sequences between *C. actinidicola* and the type strains of *C. celtidis* and *C. spicati* revealed consistent genetic differences: ITS = 0/553 (0), 1/553 (0.18%); *gapdh* = n/a, 0/238 (0); *chs-1* = 9/264 (3.4%), 1/271 (0.39%); *act* = 5/269 (1.86%), 4/269 (1.49%); *β-tubulin* = 2/400 (0.5%), 0/423 (0); *his3* = n/a, n/a; and *cal* = n/a, n/a, respectively. These molecular differences, particularly the high variation, further support the separation of *C. actinidicola* from closely related taxa. Moreover, the PHI test showed no significant evidence of recombination between *C. actinidicola* and its closest relatives, *C. celtidis* and *C. spicati* (Φw = 1.0; Fig. 2), further supporting its status as a distinct lineage. In conclusion, the combination of strong phylogenetic support, clear morphological differentiation, and genetic divergence justifies the recognition of *Colletotrichum actinidicola* as a novel species within the *C. boninense* species complex.

#### ﻿ Colletotrichum poalesicola

Taxon classificationFungiGlomerellaceae

Norph. & X.C. Wang sp. nov.

5A27EE93-320D-54FB-B2FA-3903656BFBFD

MycoBank No: 903942

Figs 4, 5

##### Etymology.

The epithet refers to “dweller on *Poales*,” utilizing the Latin suffix -cola, meaning “inhabitant” or “dweller.”

##### Type.

China • Guizhou Province, Zunyi City, Symptomatic leave of *Poaceae* sp. (bamboo), 2024.03.02, coll. Wang Xingchang, Rz1-2/GZ28-1/28-2 (dried culture, HGUP 25-0041, holotype), ex-type living culture GUCC 25-0040.

##### Description.

Isolated from bamboo. ***Sexual morph***: Not observed. ***Asexual morph*: *Conidiomata*** pycnidial, globose, dark brown, superficial on PDA, releasing conidia in a yellow mass, slimy, globose. ***Conidiophores*** either directly formed from hyphae or from a cushion of spherical hyaline cells, septate, and branched. ***Conidiogenous cells*** hyaline to pale brown, cylindrical to clavate, straight to flask-shaped, (17–)18–40(–53) × (3–)4–5(–5.8) μm (mean ± SD = 33 ± 0.9 × 4 ± 0.3 μm). ***Setae*** not observed. ***Conidia*** (12–)12.5–14(–15) × (6–)6.5–7.5(–8.5) μm (mean ± SD = 13 ± 0.8 × 7 ± 0.2 μm), n = 50, L/W ratio = 2.2, hyaline, aseptate, smooth-walled, ellipsoidal to cylindrical, one end rounded and one end acute or both ends rounded, guttulate, granular.

##### Culture characteristics.

Colonies on PDA reach 7–9 cm in diameter after 7 days at room temperature (±25 °C), exposed to 12 hours of light and 12 hours of darkness. The colonies are rhizoid to filamentous, dense, with a cottony or floccose surface and a filamentous margin. After 14 days, the colony appears white from above, producing grouped black and yellow pycnidia with orange conidial mass in the center and a white to pale-yellow reverse (Fig. 5a, b).

##### Material examined.

China • Guizhou Province, Zunyi City, Symptomatic leaves of *Poaceae* sp. (bamboo), 2024.03.02, coll. Wang Xingchang, Rz1-2/GZ28-1/28-2 (dried culture, HGUP 25-0041, holotype), ex-type living culture GUCC 25-0040, GUCC 25-0057.

##### Notes.

**Figure 4.**
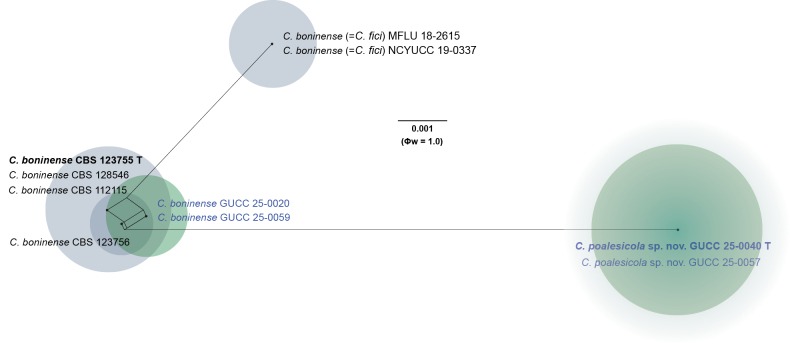
Split graph displaying the results of the pairwise homoplasy index (PHI) test of *Colletotrichum poalesicola* sp. nov and closely related taxa using Log-det transformation and splits decomposition options. Φw ≥ 0.05 from the PHI test signifies no substantial recombination in the dataset. The type species are indicated in bold, and the newly identified taxon and isolates are displayed in blue.

Phylogenetic analyses based on multi-locus sequence data place *Colletotrichum poalesicola* within the *C. boninense* species complex, forming a distinct clade closely related to *C. boninense* sensu stricto with support of 61% BSML, 51% BSMP, and 0.75 BYPP (Fig. 1). Morphologically, *C. poalesicola* can be readily distinguished from its closest relatives by the dimensions and shape of its conidia. The conidia of *C. poalesicola* are shorter and broader, measuring (12–)12.5–14(–15) × (6–)6.5–7.5(–8.5) µm, compared to those of *C. boninense*, which are more narrowly ellipsoidal [(11.5–)13–15.5(–17) × (4–)5–6(–7) µm] (Moriwaki et al. 2003). Molecular differentiation is further supported by pairwise sequence comparisons between *C. poalesicola* and the type strain of *C. boninense*, which revealed notable base pair differences across multiple loci: ITS = 1/533 (0.18%), *gapdh* = 4/251 (1.59%), *chs-1* = 27/271 (9.96%), *act* = 1/269 (0.37%), *β-tubulin* = 1/495 (0.20%), *his3* = 0/387 (0%), and *cal* = 0/424 (0%). The high level of divergence observed in the *chs-1* locus, in particular, provides strong molecular evidence for species delimitation. Moreover, results from the PHI recombination test revealed no significant recombination among *C. poalesicola* and *C. boninense* (Φw > 1.0; Fig. 4), further corroborating their genetic distinctiveness and independent evolutionary trajectory. In conclusion, the combination of robust phylogenetic support, consistent morphological differences, and significant molecular divergence justifies the recognition of *Colletotrichum poalesicola* as a novel species within the *C. boninense* species complex.

**Figure 5.**
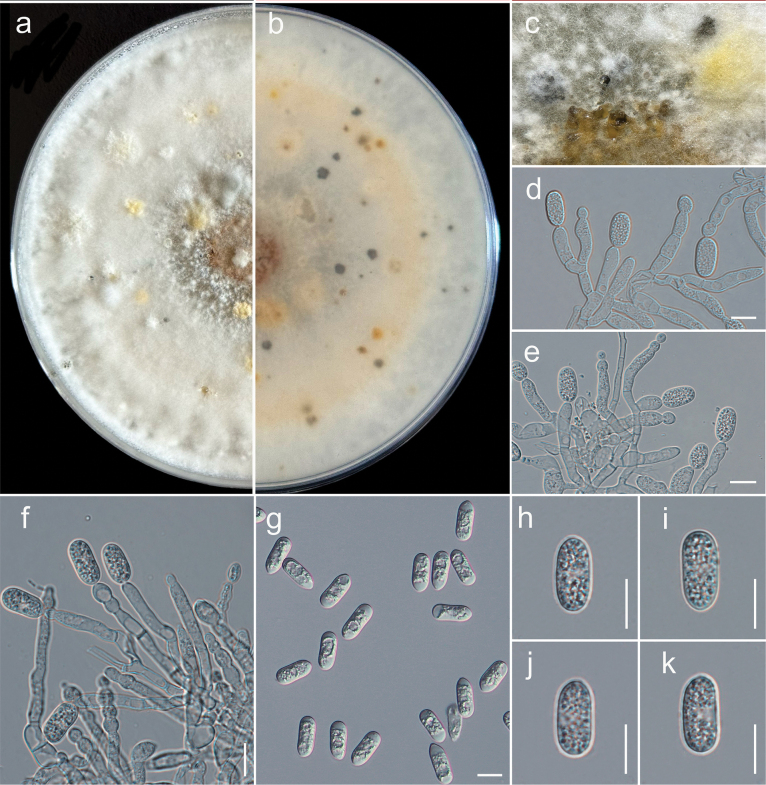
*Colletotrichum poalesicola* (GUCC 25-0040, ex-type). a, b. Culture on PDA (a-above, b-reverse); c. Conidiomata on PDA; d–f. Conidiophore, conidiogenous cells giving rise to conidia; g–k. Conidia. Scale bars: 10 µm (d–k).

#### ﻿ Colletotrichum karsti

Taxon classificationFungiGlomerellaceae

You L. Yang, Zuo Y. Liu, K.D. Hyde & L. Cai, Cryptog. Mycol. 32(3): 241 (2011)

E22200B8-0FC8-5D7F-B19E-11BBC9B747D7

MycoBank No: 842296

Fig. 6

= Colletotrichum wuxuhaiense Z.F. Yu & Hua Zheng, J. Fungi 8(1, no. 87): 20 (2022).

##### Description.

Isolated from a leaf spot of *Rosa chinensis* Jacq. ***Sexual morph***: undetermined. ***Asexual morph*: *Conidiomata*** pycnidial, globose, dark brown, superficial on PDA, releasing conidia in a yellow mass, slimy, globose. ***Conidiophores*** produce conidiogenous cells, either directly formed from hyphae or from a cushion of spherical hyaline cells, septate, and branched. ***Conidiogenous cells*** hyaline, cylindrical to clavate, straight to flask-shaped, (17.4–)18–50(–53.2) × (3.2–)4–5(–5.8) μm (mean ± SD = 33 ± 0.9 × 4 ± 0.2 μm). ***Setae*** not observed. ***Conidia*** (12.6–)13–14.8(–15) × (6.2–)6.3–7.9(–8.4) μm (mean ± SD = 14 ± 0.8 × 7 ± 0.2 μm), n = 30, L/W ratio = 2.1, hyaline, aseptate, smooth-walled, ellipsoidal to cylindrical, one end rounded and one end acute or both ends rounded, sometimes the base slightly pointed with a prominent scar, guttulate, granular. ***Appressoria*** single in short chains, pale brown, thick-walled, entire edge, rarely lobate, smooth-walled.

**Figure 6.**
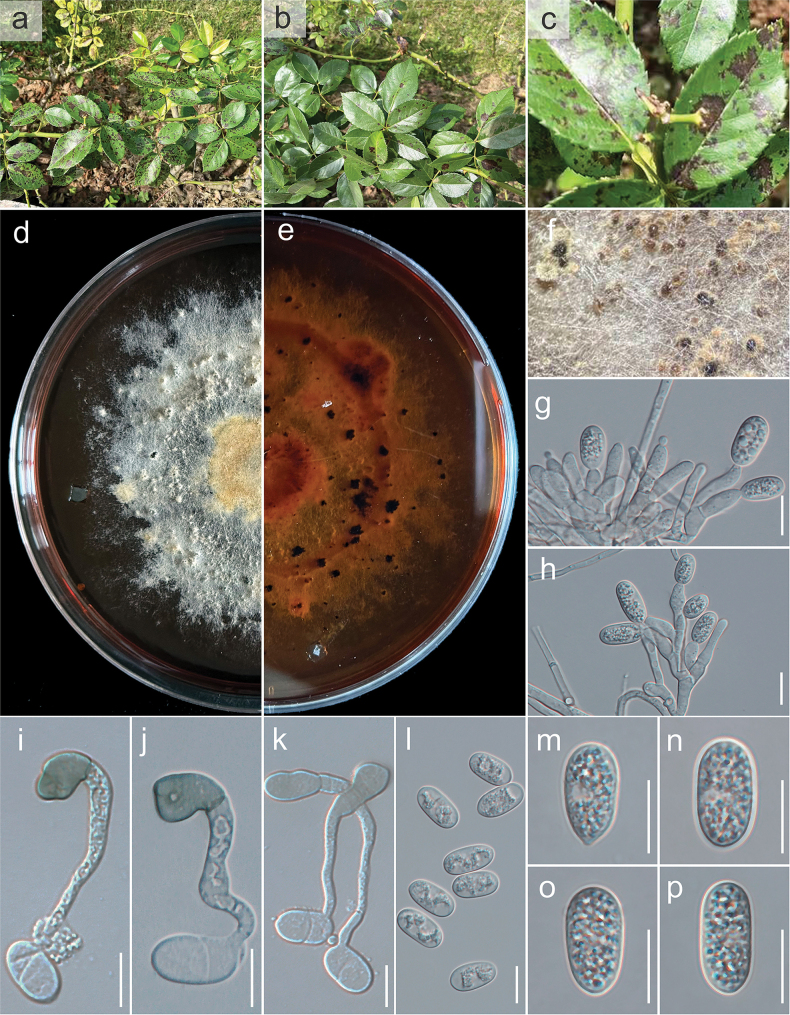
*Colletotrichum karsti* (GUCC 25-0060, new host record); a–c. *Rosa chinensis*, host and habitat; d, e. Culture on PDA (a-above, b-reverse); f. Conidiomata on PDA; g, h. Conidiophore, conidiogenous cells giving rise to conidia; i–k. Appressorium; l–p. Conidia. Scale bars: 10 µm (g–p).

##### Culture characteristics.

Colonies on PDA reach 5 cm in diameter after 7 days at room temperature (±25 °C), exposed to 12 hours of light and 12 hours of darkness. The colonies are rhizoid to filamentous, dense, with a filamentous margin. After 14 days, the colony appears white to pale-yellow from above, producing grouped dark brown pycnidia and a white to yellow reverse (Fig. 5a, b).

##### Material examined.

China • Yunnan province, Kunming, Kunming Botanical Garden, Symptomatic leaves of *Rosa chinensis* Jacq., 2023.10.29, Wang Xingchang, KM004-1/GZ25-1/GZ25-2 (dried culture, HGUP 25-0042), living culture GUCC 25-0060, GUCC 25-0037.

##### Notes.

Based on multi-locus phylogenetic analyses (Fig. 1), *Colletotrichum wuxuhaiense* was found to cluster within the *Colletotrichum karsti* species clade, with strong statistical support (82% BSML, 74% BSMP, and 1.00 BYPP). Morphologically, both species exhibit similar features, including cylindrical to clavate, hyaline conidiophores and ellipsoidal to cylindrical, one-celled, smooth-walled, hyaline conidia that are straight, truncate at the base, and obtuse at the apex. Despite their overall similarity, minor differences were observed in conidial size: *C. wuxuhaiense* (YMF 1.04951) measured 10.3–15.8 × 4.7–8.9 µm, whereas *C. karsti* (CBS 132134) ranged from 12.5–19.5 × 6.0–8.5 µm. Additionally, *C. wuxuhaiense* was characterized by a prominent basal scar and conidiogenous cells with distinct periclinal thickening (Yang et al. 2011; Zheng et al. 2022). Pairwise comparisons between the type strains of *C. wuxuhaiense* (YMF 1.04951) and *C. karsti* (CBS 132134) revealed very low genetic divergence across five gene regions: ITS = 0.2% (1/503 bp, including one gap), gapdh = 2% (4/198 bp, including one gap), chs-1 = 0.4% (1/234 bp, including one gap), act = 2% (5/241 bp, including two gaps), and tub2 = 0.4% (2/470 bp, zero gaps). The strong phylogenetic affinity and low sequence variation across multiple loci indicate that these taxa represent the same species. Therefore, based on both molecular and morphological evidence, *C. wuxuhaiense* (Zheng et al. 2022) is regarded as a later synonym of *C. karsti* (Yang et al. 2011), in accordance with the International Code of Nomenclature for algae, fungi, and plants (ICN).

In this study, Strains GUCC 25-0060 and GUCC 25-0037, isolated from symptomatic leaves of *Rosa chinensis* (Rosaceae) in Kunming, Yunnan Province, China, were identified as *Colletotrichum karsti* based on multi-locus phylogenetic analyses. These strains clustered within the *C. karsti* clade with moderate to strong statistical support (86% BSML, 75% BSMP, and 1.00 BYPP) (Fig. 1). Their morphological features are consistent with the original description of *C. karsti*, further supporting their identification (Yang et al. 2011). *Colletotrichum karsti* was initially described from a leaf of *Vanda* sp. collected in Guizhou Province, China (Yang et al. 2011), and is now recognized as both an endophyte and a pathogen across a wide range of plant hosts (Damm et al. 2012; Zheng et al. 2022). The present study represents the first record of *C. karsti* associated with *Rosa chinensis*, thus expanding the known host range of this species.

#### ﻿ Colletotrichum boninense

Taxon classificationFungiGlomerellaceae

Moriwaki, Toy. Sato & Tsukib., Mycoscience 44(1): 48 (2003)

42837543-FD3B-54DD-B31D-60DDE06AB25E

= Colletotrichum fici Tennakoon, C.H. Kuo & K.D. Hyde, Fungal Diversity 108: 158 (2021)

##### Material examined.

China • Yunnan Province, Dali, Symptomatic fruit of *Juglans regia* L., 2023.11.12, Zhou Mengting, DJ16-7/GZ01/GZ01-2 (dried culture, HGUP 25-0044), living culture GUCC 25-0020, GUCC 25-0059.

##### Hosts and distribution.

Damm et al. (2012), Zhang et al. (2024).

##### Notes.

In the phylogenetic tree (Fig. 1), *C. fici* (strains MFLU 18-2615, NCYUCC 19-0336, and NCYUCC 19-0337) clusters within the *C. boninense* s.str. clade. When *C. fici* was originally described, the study lacked comprehensive taxon sampling within the *C. boninense* complex and included only the type strain of *C. boninense* s.str. Additionally, the molecular data for *C. fici* were limited to ITS, *act*, and *chs-1* loci (Tennakoon et al. 2021). Pairwise comparisons between *C. fici* and the type strain of *C. boninense* s.str. showed less nucleotide differences: ITS = 1/553 (0.2%), *act* = 2/269 (0.74%), and *chs-1* = 1/245 (0.4%). These differences fall within the range of intraspecific variation. Therefore, based on both phylogenetic and molecular evidence, *C. fici* is herein regarded as a synonym of *C. boninense* s.str. Additionally, in this study, strains GUCC 25-0020 and GUCC 25-0059, isolated from *Juglans regia* in Kunming, China, were identified as *Colletotrichum boninense* based on phylogenetic analysis (Fig. 1), with strong phylogenetic support (99% BSML, 0.98 PPBI).

## ﻿Discussion

The genus *Colletotrichum* represents one of the most important groups of phytopathogenic fungi, known for causing anthracnose and other destructive plant diseases across a wide range of economically significant hosts worldwide (Dean et al. 2012). Within this genus, the *C. boninense* species complex comprises numerous morphologically similar but genetically distinct taxa, often associated with both cultivated and wild plants (Damm et al. 2012; Marin-Felix et al. 2017; Jayawardena et al. 2021; Talhinhas and Baroncelli 2021). Molecular data have become essential for resolving taxonomic ambiguities in *Colletotrichum* (Marin-Felix et al. 2017; Bhunjun et al. 2021; Jayawardena et al. 2021; Liu et al. 2022). Multilocus phylogenetic analyses using markers such as ITS, *gapdh*, *act*, *chs-1*, *β-tubulin*, *cal*, *his3*, and *ApMat* have significantly improved species resolution, especially within the *C. gloeosporioides*, *C. acutatum*, and *C. boninense* complexes (Weir et al. 2012; Jayawardena et al. 2021; Liu et al. 2022). These methods are crucial for distinguishing cryptic species, correcting misidentifications, and resolving invalid taxa, particularly in a genus exhibiting high morphological plasticity.

This study makes a significant contribution to the taxonomy and ecology of *Colletotrichum* species in southwestern China by resolving species boundaries using an integrated approach that combines morphological and multilocus phylogenetic data. All eight strains analyzed belong to the *C. boninense* species complex, a group known for its phylogenetic complexity and cryptic diversity (Damm et al. 2012; Weir et al. 2012). Two novel species, *C. actinidicola* and *C. poalesicola*, are described herein based on distinct phylogenetic placements and morphological characteristics, including differences in conidial size, colony appearance, and appressorial morphology. Multilocus phylogenetic analyses (ITS, *gapdh*, *chs-1*, *act*, *β-tubulin*, *his*, *cal*) robustly resolve these taxa as separate lineages. Their taxonomic status is further corroborated by the genealogical concordance phylogenetic species recognition (GCPSR) approach, providing strong evidence for their delimitation as independent species within the *C. boninense* species complex. These discoveries are consistent with recent mycological surveys in Southeast Asia, India, and China, where numerous *Colletotrichum* taxa have been delineated from diverse host plants using similar integrative approaches (Maharachchikumbura et al. 2016; Bhunjun et al. 2021; Zakaria 2021; Zheng et al. 2022; Zhang et al. 2023a, b, 2024). Collectively, these findings highlight the rich yet still underexplored fungal diversity of tropical and subtropical Asia.

The synonymy of *Colletotrichum fici* with *C. boninense* is supported by strong phylogenetic evidence, as strains previously identified as *C. fici* consistently grouped with the *C. boninense* clade without significant divergence. This supports earlier suggestions by Weir et al. (2012) and Chen et al. (2022), who noted that phylogenetically related strains often share similar secretome profiles, although the overall composition may differ across species complexes. These findings indicate that *C. fici* may not represent a distinct species. Likewise, the synonymy of *C. wuxuhaiense* with *C. karsti* is justified by minimal genetic differences and overlapping morphological characteristics. Such taxonomic clarification is essential for reducing redundancy and confusion in fungal systematics. Moreover, *C. karsti* was isolated for the first time from *Rosa chinensis* in China, representing a new host record for this species. This finding extends the known host range and geographical record in China of *C. karsti*, which was previously reported on *Juglans regia* and *Ginkgo biloba* (Li et al. 2024; Wang et al. 2025). Notably, a recent report from South Africa also identified *C. karsti* on *Rosa* leaves exhibiting anthracnose symptoms (Maguvu et al. 2024), suggesting a potential global emergence of this species on ornamental hosts. The recent availability of the *C. karsti* genome further enhances its importance by enabling comparative genomic studies to investigate pathogenicity, host adaptation, and evolutionary dynamics. These findings collectively emphasize the need for heightened surveillance of *C. karsti*, particularly in ornamental and horticultural systems, where its broad host adaptability poses a potential threat to plant health and biosecurity (Jayawardena et al. 2016).

These findings have direct implications for plant disease management in the region. Species in the *C. boninense* complex are associated with a wide variety of hosts and are often involved in anthracnose diseases that can affect economically important crops such as citrus, avocado, tea, watermelon, and ornamentals (Cannon et al. 2012; Schena et al. 2014; Hyde et al. 2014; Guo et al. 2022). Accurate species identification is critical for developing targeted control strategies, as pathogenicity, fungicide sensitivity, and host specificity may vary significantly among closely related species (O’Connell et al. 2012; Liu et al. 2015, 2022; Talhinhas and Baroncelli 2021). For instance, misidentification of species could lead to ineffective disease control recommendations or misinterpretation of host range and epidemiology (Jayawardena et al. 2021; Talhinhas and Baroncelli 2021; Liu et al. 2022). Furthermore, the present study highlights the importance of ongoing mycological exploration in biodiverse regions, such as Guizhou and Yunnan provinces. Despite increased fungal surveys in Asia, many habitats remain underexplored, and it is likely that additional novel species and new host records remain to be discovered (Liu et al. 2015; Hyde et al. 2020, 2024; Wijayawardene et al. 2021, 2022; Zheng et al. 2022; Zhang et al. 2023a, 2024; Jayasiri et al. 2024).

In conclusion, this study describes two new *Colletotrichum* species, clarifies the taxonomy of two previously ambiguous species, and expands the known host range of *C. karsti*. These findings enhance our understanding of *Colletotrichum* biodiversity in Asia and contribute valuable data for future research on fungal systematics, plant pathology, and biodiversity conservation.

## Supplementary Material

XML Treatment for Colletotrichum actinidicola

XML Treatment for Colletotrichum poalesicola

XML Treatment for Colletotrichum karsti

XML Treatment for Colletotrichum boninense

## ﻿Additional information

### Use of AI

No use of AI was reported.

### Author ORCIDs

Chada Norphanphoun https://orcid.org/0000-0002-5756-7206

Jia-Ping Wang https://orcid.org/0009-0002-7558-4760

Xing-Chang Wang https://orcid.org/0009-0008-4913-5316

Herbert Dustin Aumentado https://orcid.org/0000-0003-2888-2669

Yong Wang https://orcid.org/0000-0003-3831-2117

### Data availability

All of the data that support the findings of this study are available in the main text.
